# Inoculation for Typhoid Fever

**Published:** 1900-01-27

**Authors:** 


### INOCULATION FOR TYPHOID FEVER.

Typhoid fever is so important a factor in war, and the possibility of an outbreak of this disease in epidemic form is so imminent a peril to the troops now engaged in South Africa, that the publication by Professor Wright and Major Leishman, R.A.M.C., of the results which have followed their anti-typhoid inoculations are of the greatest possible interest at the present moment. The inoculations in question were mostly performed in the end of the year 1898 and the beginning of the year 1899, among British troops in India. The material employed was a culture of virulent typhoid bacilli, which, after being allowed to grow for a given time, had been sterilised by exposure to a temperature of 60 deg. C., or in some cases to a somewhat higher temperature. Thus the process differs both from Jennerian vaccination, in which the virulence of the micro-organism has been attenuated by its being passed through a series of appropriate animals, and also from the Pasteurian method of vaccinating against anthrax, in which the micro-organisms are artificially attenuated, for in the anti-typhoid vaccine the microbes are not attenuated but killed. The method now in question for "vaccination" against typhoid consists in the inoculation of the patient with measured quantities of dead but still poisonous typhoid bacilli, the quantity given being such as to set up the largest degree of immunity which can be obtained without producing a dangerous amount of immediate reaction. In view of the statements recently made to the effect that this sterilisation is not in all cases complete, and that the vitality of the bacillus is merely lowered, so that after all the patient goes through what may be called a modified attack of typhoid fever, it must be pointed out that, according to the papers by Drs. Wright, Semple, and Leishman, in which the proceeding is described, it is definitely laid down that it is a sterilised culture that is used, one of the great objects of using such dead vaccines being that of avoiding the possibility of producing typhoid fever by their use. The reaction may be severe, and the patient may be made ill, but he is not made to suffer from typhoid fever. Now for the results. Taking the tables, which are given as they stand, it appears that a total of 11,295 individuals were under observation for the purposes of the experiment. Of these 2,835 were inoculated, while 8,460 were not inoculated. So far as information had been received in regard to the after history of these cases it appears that among the inoculated 27 cases of typhoid fever had arisen, or at the rate of 0·95 per cent., while among those not inoculated 213 cases of typhoid had occurred, or a percentage of 2·5. Thus the proportion of cases among those who had not been inoculated was nearly three times as large as the proportion occurring in the same time among the inoculated. This is a very satisfactory result, and all the more so when we look into the details, for it appears that the cases which were inoculated comprised a considerable number of young and unseasoned soldiers who, as is well known, are far more likely to be attacked by typhoid fever on arrival in India than are the older men and those who have been longer in the country. Again, it is clear that the conditions under which the inoculations were carried out, the frequent transportation of the cultivations from place to place, and the measures taken for their preservation by repeated reheating were by no means calculated to produce the best possible results. When, then, we consider how well the experiment turned out, even under such difficult circumstances, we can hardly doubt this form of inoculation will be found of distinct service, and that it will be found possible by its means to give at least a temporary protection against typhoid fever to those who are compelled to expose themselves to risks of infection. How long this immunity will last is a question to which no answer seems at present possible, but it seems fair to believe that for 18 months or a couple of years the protective influence may be more or less relied upon. Do not let anyone imagine that universal inoculation against typhoid fever is a thing to be advised or to be looked forward to for any nation's good. So far as the ordinary circumstances of life are concerned we have better ways of combating typhoid fever than by inoculation, and no country should be satisfied with the stage of civilisation to which it has attained unless this gives it a fair protection against this disease—this punishment for uncleanly living. But war is another matter. War upsets everything, and so ghastly an addition even to the horrors of war is typhoid fever, and so enormously does its occurrence hamper the efficiency of any army, that although we hold ourselves quite aloof from those who would rush in with the inoculating syringe for the control of such a disease in ordinary circumstances, we would certainly urge those who are about to expose themselves to the risks of war to undergo a proceeding which, although a little irksome, seems harmless, and may save them from many risks, while saving the army of which they are a part from many disasters.

